# Computerised cognitive–behavioural therapy for depression in adolescents: 12-month outcomes of a UK randomised controlled trial pilot study

**DOI:** 10.1192/bjo.2019.91

**Published:** 2019-12-12

**Authors:** Barry Wright, Lucy Tindall, Rebecca Hargate, Victoria Allgar, Dominic Trépel, Shehzad Ali

**Affiliations:** Chair in Child Mental Health, Hull York Medical School, University of York & Leeds and York Partnership NHS Foundation Trust, UK; Research Fellow, Leeds and York Partnership NHS Foundation Trust, UK; Research Manager, Leeds and York Partnership NHS Foundation Trust, UK; Professor in Medical Statistics, Hull York Medical School, University of York, UK; Health Economist, Department of Health Sciences, University of York, UK; Health Economist, Department of Health Sciences, University of York, UK

**Keywords:** Randomised controlled trial, computerised cognitive behaviour therapy, depression, adolescents, twelve month outcomes

## Abstract

**Background:**

Computerised cognitive–behavioural therapy (CCBT) in the care pathway has the potential to improve access to psychological therapies and reduce waiting lists within Child and Adolescent Mental Health Services, however, more randomised controlled trials (RCTs) are needed to assess this.

**Aims:**

This single-centre RCT pilot study compared a CCBT program (Stressbusters) with an attention control (self-help websites) for adolescent depression at referral to evaluate the clinical and cost-effectiveness of CCBT (trial registration: ISRCTN31219579).

**Method:**

The trial ran within community and clinical settings. Adolescents (aged 12–18) presenting to their primary mental health worker service for low mood/depression support were assessed for eligibility at their initial appointment, 139 met inclusion criteria (a 33-item Mood and Feelings Questionnaire score of ≥20) and were randomised to Stressbusters (*n* = 70) or self-help websites (*n* = 69) using remote computerised single allocation. Participants completed mood, quality of life (QoL) and resource-use measures at intervention completion, and 4 and 12 months post-intervention. Changes in self-reported measures and completion rates were assessed by group.

**Results:**

There was no significant difference between CCBT and the website group at 12 months. Both showed improvements on all measures. QoL measures in the intervention group showed earlier improvement compared with the website group. Costs were lower in the intervention group but the difference was not statistically significant. The cost-effectiveness analysis found just over a 65% chance of Stressbusters being cost-effective compared with websites. The 4-month follow-up results from the initial feasibility study are reported separately.

**Conclusions:**

CCBT and self-help websites may both have a place in the care pathway for adolescents with depression.

## Background

The 1-year prevalence of depression in adolescents is estimated to be between 2 and 4%^1,2^ and is one of the most common mental health problems facing young people.^3^ Given the importance of early treatment, recent government priority has focused on improving access to psychological therapies including cognitive–behavioural therapy (CBT). Reviews of CBT for adolescent depression have shown that it is effective and currently one of the main treatment options recommended for this group.^4,5^ Despite this, the availability of effective mental health treatments with Child and Adolescent Mental Health Services (CAMHS) is limited, with limited staff numbers, long waiting times and severity access thresholds needed to receive treatment. In a recent UK study waiting times for emotional and behavioural problems was 20 weeks (95% CI 18–22 weeks).^6^ In this study of 21 000 referred children, only 72% referrals were accepted, and the most common group being rejected was those with emotional and behavioural problems.

## Stressbusters

A potential solution to this problem is the delivery of CBT in a computerised form (CCBT). It is easily accessible and addresses some young people's reluctance to access mental health services. CCBT may reduce this barrier in providing easy access in the community, without need for regular face-to-face contact. Several CCBT programs have shown effectiveness in well-designed randomised controlled trials (RCTs), however, replication and long-term follow-up studies are needed to confirm results.^7^ An example of CCBT for the treatment of adolescent depression is Stressbusters, which has been developed in the UK. Stressbusters has shown positive results in a case series of 23 adolescents^8^ where 95% of a UK adolescent sample met diagnostic criteria for depression at baseline, falling to 22% post-treatment. This, alongside good completion rates (70% completing all eight sessions) suggests that Stressbusters is a potentially effective CCBT package warranting further investigation within a RCT. In a more recent RCT of 112 young people with depression, the effectiveness of Stressbusters was examined in a school setting,^9^ although follow-up was only for 6 months. This showed a significant reduction in adolescent depression and anxiety compared with a waiting list control as measured using the Mood and Feelings Questionnaire (MFQ)^10^ and the Screen for Child Anxiety Related Disorders. More research is required to examine the effectiveness of CCBT in comparison with an attention control to ensure any effects observed are a result of the CCBT intervention.

## Aims

At the time of the current study there were no trials of CCBT for the treatment of depression in adolescents. Given the promising results from the initial evaluation of the Stressbusters programme^8^ this feasibility and pilot study aimed to evaluate the clinical effectiveness of Stressbusters with adolescents with low mood/depression.

## Method

We examined the clinical and cost-effectiveness of Stressbusters by comparing it to an attention control (self-help websites) within a RCT. This comprised an initial feasibility RCT followed by a pilot RCT (trial registration: ISRCTN31219579).

The first phase of this research was a Research for Patient Benefit-funded study examining the feasibility of recruiting to CCBT and delivering it as a treatment to adolescents with low mood. Overall 97 young people were recruited across a 24-month recruitment period. The results of the 4-month follow-up from the initial feasibility study are reported separately).^11^ Following successful recruitment and good feasibility outcomes (for example acceptability of outcome measures, interventions and involvement in an RCT^11^) in the first phase we applied for a 12-month extension in order to continue into a second phase of recruitment, the pilot study. As part of this second phase recruitment continued without a break to 139 young people following the same protocol. The results reported are of the combined analysis of outcome measures between both recruitment phases.

### Participants and recruitment

Our target population was 12- to 18-year-olds with low mood/depression living within the areas covered by a CAMHS service in a Northern City in England.

The study was conducted between June 2011 and August 2016. Initially 97 young people were recruited between June 2011 and May 2013 with a further 48 recruited up until April 2015 when the trial recruitment ended. Trial referrals were made by nine primary mental health workers (PMHWs) covering a 300 000 population catchment area, who screened adolescents referred to them with low mood/depression using the MFQ^10^ at their initial assessment appointment. The MFQ is a 33-item questionnaire, based on DSM-III-R criteria for depression, comprising descriptive phrases regarding how an individual has been feeling or behaving in the preceding 2 weeks.^10^

Eligibility was defined by a score of ≥20 on the MFQ^10^ (validation research^12^ proposes a score of ≥20 indicates any depressive disorder and ≥29 a likely current major depressive episode). A Cronbach's alpha of 0.95 for the MFQ has been reported^12^ suggesting high internal consistency. Daviss *et al* (2006) examined the criterion validity of child- and parent-versions of the MFQ (the MFQ-C, MFQ-P, respectively) in a heterogeneous sample of children and adolescents from clinic and non-clinic origins.^12^ The authors found that a score of 29 on the MFQ-C (positive screen rate 21%, sensitivity 68%, specificity 88%) or 27 on the MFQ-P (positive screen rate 23%, sensitivity 61%, specificity 85%) optimally discriminated youth with major depressive episodes from the rest of the sample.

Young people gave fully informed consent (with that of their parent/guardian if under 16). Those with comorbid physical illness were offered the trial if eligible, however, those with psychosis or active suicidality were not. Clinicians decided upon treatment pathways in discussion with young people and their families. Those with severe depression were referred to the local CAMHS team for further assessment and treatment in the first instance.

All young people entering the trial could access additional ‘treatment as usual’ at any point if this was deemed necessary. On their completion or withdrawal from the study all young people were referred back to their PMHW who assessed whether any additional treatment was required.

### Ethics

The trial was designed to protect the human rights and dignity of the participant as reflected in the 1996 version of the Helsinki Declaration. Ethical approval for this trial was received by Leeds (West) Research and Ethics Committee (Reference: Phase one: 10/H1307/137, Phase two: 14/YH/0045).

### Outcome measures

Once consented, participants completed the following baseline measures.

#### Short Beck Depression Inventory (BDI) (primary outcome measure)

The short BDI is a 13-item self-report measure used to assess depression severity among adolescents by measuring cognitive, behavioural, affective and somatic dimensions of depression.^13^

#### Spence Children's Anxiety Scale (SCAS)

The SCAS is a 45-item, self-report measure used to assess the severity of anxiety within six subgroups (generalised anxiety, panic/agoraphobia, social phobia, separation anxiety, obsessive compulsive disorder and physical injury fears) with an overall anxiety score.^14^ Internal consistency shows^15^ a coefficient alpha of 0.92 and a Guttman split half reliability of 0.90.

#### Quality of life (QoL), including the EuroQol EQ-5D-Y and Health Utility Index Mark 2 (HUI-2) and service use questionnaire

Self-report questionnaires were used to obtain information about health-related QoL and service use. This comprised: (a) the EuroQol EQ-5D-Y^16^ and (b) the HUI-2,^17^ both instruments were used for capturing health-related QoL in young people; and (c) a service use questionnaire to capture patient-level service use. The service use questionnaire was a bespoke measure developed for use in the study by the health economists in consultation with the wider study team.

#### Preference scale (treatment allocation)

A preference scale was used to determine participant preference for the trial arm they would have preferred to complete if the research did not involve randomisation, this was completed at baseline. This was a visual analogue scale (0–100) with Stressbusters (100), websites (0) or no preference (50). Participants were informed that responses on this scale would have no impact on trial allocation.

#### Demographic information

Participants were asked to supply demographic information including their age, gender and ethnicity, their education and employment status and their family life and social relationships.

### Randomisation

Following the completion of baseline measures participants were randomised to one of two trial arms using remote computerised single allocation (provided by the University of York Trials Unit).

### Arm one: CCBT intervention: ‘Stressbusters’

Stressbusters is a CCBT program comprising eight 30–45 min sessions of CBT designed for 12- to 18-year-olds. The program is based on the manualised treatment program from an RCT designed to evaluate the effectiveness of CBT compared with a placebo control.^18^ Each Stressbusters session is an interactive presentation featuring videos, animations, graphics and printouts. These are completed in linear succession with each building on knowledge gained in previous sessions. Homework tasks included problem-solving and mood diaries, with young people recording relevant information on the computer at their next session. Sessions contained flexible ‘add-ons’ such as fact sheets (for example about bullying, sleep problems) and practice-related hand-outs.

Video inserts (case vignettes) of three teenagers feature throughout. Participants hear about the lives of the teenagers through a combination of short video sequences and voiceovers. The participant inputs information (such as mood ratings and activity plans), which is stored and used throughout.

The content is organised as follows:

- session 1: introduction and goal setting;
- session 2: getting activated;
- session 3: emotion recognition;
- session 4: noticing thoughts;
- session 5: thought challenging;
- Session 6: problem-solving;
- session 7: improving social skills;
- session 8: relapse prevention.

### Arm two: attention control – self-help websites

Participants in arm two spent equivalent time accessing currently available self-help websites. These were chosen by an expert clinical panel, with user and carer involvement, based on their suitability for use with the participant age range, having positive self-help well-being and mood advice and having minimal CBT content. All selected websites provided helpful information about low mood/depression in a combination of texts, narratives and videos. Participants were instructed to navigate freely through the website they were looking at and were able to decide what content they viewed and in what order. There were no homework assignments given to those randomised to the self-help website arm of the trial.

The sites were: www.youngminds.org.uk; http://www.depressioninteenagers.com; www.RU-OK.org.uk; www.healthtalk.org.

Participants were introduced to a new website at each of the first four sessions. After introduction of all four websites they could spend subsequent sessions returning to the sites/areas that they found most helpful.

### Procedure

A researcher (either a National Health Service (NHS) band 5 research assistant or an NHS band 6 clinical studies officer, educated to a minimum of degree level with experience in mental health research) met individual participants at each session. The researcher greeted the participant and provided them with information about the session format before leaving them alone to either access Stressbusters or a self-help website. The researcher remained in close proximity for the duration of the session to answer any questions and provide any practical support with the computer if required. The researcher did not provide any therapy. Participants were offered a choice of venue to complete trial sessions; including their school, CAMHS site, general practice surgery or community centre. All sites provided private spaces that protected confidentiality. Sessions were typically once per week with flexibility offered to fit around participants' other commitments. The methodology is described in more detail elsewhere.^19^

Participants completed the short version (15 items) of the MFQ^20^ at the beginning of each session to monitor mood and assess risk as adopted in the Abeles *et al* (2009)^8^ study. If a participant responded ‘true’ to the question ‘I thought about killing myself’ on the short MFQ their PMHW/CAMHS clinician was contacted immediately and asked to speak with the individual to discuss their response. If a PMHW/CAMHS clinician was unavailable this request was made to the duty clinician. Where none of the above could be contacted the individual was advised to contact their general practitioner. (This procedure also applied if a participant selected ‘true’ to the same question on the full MFQ or selected the response ‘I would like to kill myself’ or ‘I would kill myself if I had the chance’ during any completion of the BDI). No serious untoward incidents took place during the trial. Young people could access any care as usual in both arms throughout the trial.

### Follow-up period

The MFQ, BDI, SCAS and QoL/service use questionnaire were then subsequently completed at 4 and 12 months after completion/withdrawal from the intervention (results of the 4-month follow-up from the initial feasibility study are reported separately).^11^

### Sample size

In this feasibility study, if the MFQ were the primary outcome measure, to detect a difference of 8.4 points (pooled s.d. = 13.37) (the difference in change score detected on the MFQ across both groups in this study), at 80% power and 5% significance, 41 participants would be required per arm. Based on a 60% completion rate, this represents 68 per group (a total of 136 participants). Research suggests that the size of a pilot trial should be related to the size of the future definitive RCT.^21^ For such a trial designed with 90% power and two-sided 5% significance, pilot trial sample sizes for each treatment arm of 75, 25, 15, and 10 for standardised effect sizes that are extra small (0.1), small (0.2), medium (0.5), or large (0.8), respectively, are recommended. Hence, the extended recruitment period in this study allowed detection of a small effect size in the outcome measures.

### Data analysis

Data is reported in line with the Consolidated Standards Of Reporting Trials (CONSORT) statement. All participant baseline data are summarised descriptively by group and by phase. Continuous measures are reported using summary statistics (mean, s.d., median, minimum, maximum).

Comparisons were made between groups ‘as randomised’ in the primary analysis on an intention-to-treat basis. The primary analysis compared scores on the MFQ, BDI and SCAS scales between groups using a covariance pattern mixed model, where effects of interest and baseline covariates are specified as fixed effects, and the correlation of observations within patients over time is modelled by a covariance structure. The outcome modelled is total score at 4 and 12 months’ follow-up. The model included as fixed effects: randomisation group, time and randomisation group × time interaction terms. Different covariance structures for the repeated measurements, that are available as part of Stata v13, were explored and the most appropriate pattern used for the final model. Diagnostics including Akaike's information criterion^22^ were compared for each model (smaller values are preferred). Participants were only included in the model if they provided full data at baseline and outcome data for at least one post-randomisation time point (4 or 12 months). Estimates of the difference between treatment groups in scores was derived at both time points with 95% CIs and *P*-values.

### Health economic analysis

Cost-effectiveness analysis was conducted from the health services perspective over a 12-month time horizon. The key outcome was the incremental cost-effectiveness ratio (ICER), which is a ratio of the difference in costs (incremental costs) and difference in quality-adjusted life years (incremental QALYs), between the Stressbusters and website groups. Costs were based on use of the following health services between baseline and 12-month post-treatment: appointments with general practitioner, nurse (including mental health nurse), mental health worker, social worker, family therapist, occupational therapist, psychologist, psychotherapist, counsellor, psychiatrist and visits to accident and emergency departments and in-patient hospital admissions. In addition, the cost of the Stressbuster intervention (£101.27) was added for children randomised to this group. Resource use was multiplied by unit costs obtained from the Personal and Social Services Resource Use^23^ unit cost database (2016) to obtain individual-level costs.

QoL was measured using the EQ-5D-Y questionnaire at baseline, 4 months post-treatment and 12 months post-treatment. The EQ-5D-Y questionnaire was used to evaluate QoL and responses were converted to utility values (range 0, death to 1, best health) using the UK population tariff. Following this, QALYs were calculated using the area-under-the-curve approach.

The incremental difference in costs and QALYs between groups was estimated using the seemingly unrelated regression model, which takes account of the correlation between costs and QALYs. The QALY regression controlled for baseline utility.^24^ The regression coefficient on the Stressbuster group variable represented the incremental difference in costs and QALYs and the ratio of coefficients provided the ICER. The CI around the ICER was estimated using 10 000 non-parametric bootstrap replicates.^25^ The probability of the intervention being cost-effective over a range of willingness to pay (WTP) thresholds per QALY was presented using the cost-effectiveness acceptability curve (CEAC).^26^

## Results

**Fig. 1:**
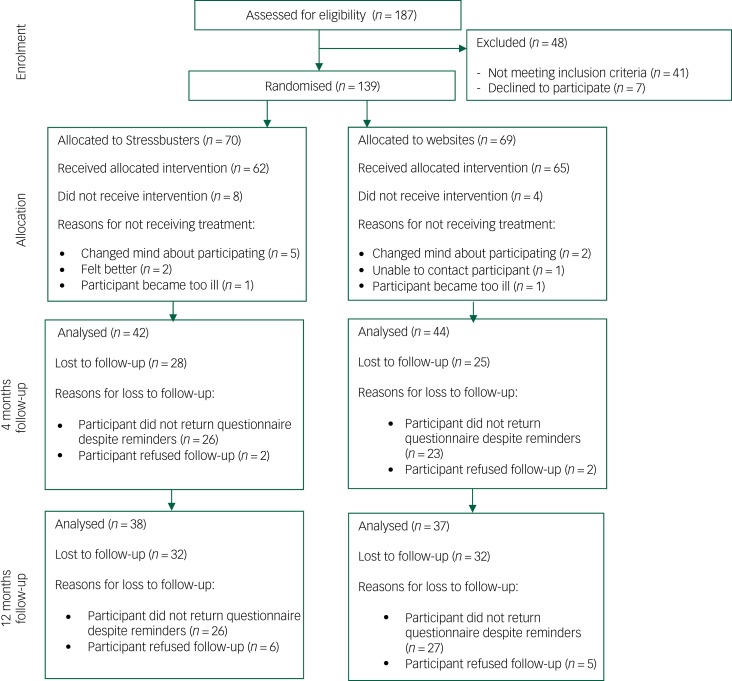
CONSORT flow diagram.

Overall 187 individuals were assessed for eligibility; 41 did not meet the inclusion criteria and 7 declined to participate. In total, 139 young people consented and were randomised to either Stressbusters (*n* = 70) or websites (*n* = 69) (See consort diagram in Fig. 1).

### Questionnaire completion

All participants completed the baseline questionnaire. At 4 months post-intervention, self-report questionnaires were sent to 65/70 (92.9%) participants in the Stressbusters group and 65/69 (94.2%) of participants in the website group. The 9 participants who did not receive a follow-up questionnaire had withdrawn completely from the trial. The overall return rate was 64.6% (42/65) for the Stressbusters group and 67.7% (44/65) for the website group.

At 12 months post-intervention, self-report questionnaires were sent to 64/70 (91.4%) participants in the Stressbusters group and 63/69 (91.3%) of participants in the website group. The 12 participants who did not receive a follow-up questionnaire had withdrawn completely from the trial. The overall return rate was 59.4% (38/64) for the Stressbusters group and 58.7% (37/63) for the website group. Overall 66 participants completed all outcome measures at both 4 and 12 months’ follow-up.

### Baseline characteristics

Table 1 shows the baseline characteristics. The Stressbuster and website groups were similar on most characteristics, although more individuals in the websites group had physical health problems (26%) than the Stressbusters group (11%) and a higher proportion in the Stressbusters group (9%) had been prescribed antidepressants compared with the websites group (3%).

**Table 1:** Characteristics of the participants at baseline by phase and total recruitment

	Stressbusters (*n* = 70)	Websites (*n* = 69)
Age at baseline, mean (s.d.)	14.9 (1.5)	15.1 (1.3)
Gender, male: *n* (%)	23 (33)	27 (39)
Ethnic group, White: *n* (%)	69 (99)	67 (97)
Do you consider yourself to have any physical health problems, yes: *n* (%)	8 (11)	18 (26)
Have you ever been bullied, *n* (%)		
I am currently	3 (4)	8 (12)
I have been in the past	49 (70)	42 (62)
I have never been bullied	18 (26)	18 (26)
Do you drink alcohol, *n* (%)		
Never	31 (44)	37 (54)
Occasionally	37 (53)	31 (45)
Frequently	2 (3)	1 (1)
Have you previously experienced any episodes of low mood, yes: *n* (%)	66 (94)	63 (91)
Do you seek help to deal with your low mood, yes: *n* (%)	47 (67)	46 (67)
Have you ever been prescribed antidepressants, *n* (%)		
Yes	6 (9)	2 (3)
No	42 (60)	48 (70)
Not applicable	22 (31)	19 (28)
Cognitive–behavioural therapy in the past, yes: *n* (%)	16 (23)	15 (22)
Counselling in the past, yes: *n* (%)	21 (30)	29 (42)
Other talking therapies in the past, yes: *n* (%)	5 (7)	2 (3)

### Outcome measures

**Fig. 2:**
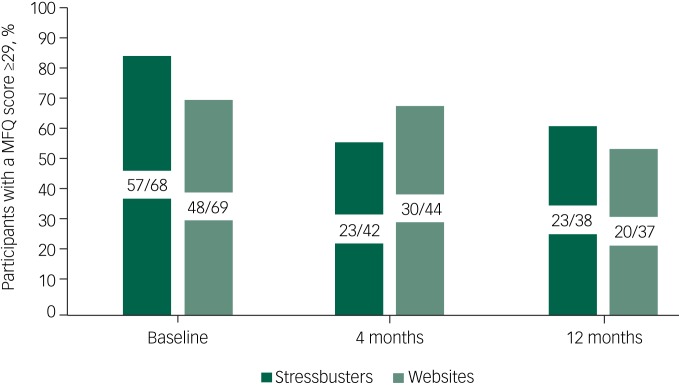
Proportion who scored ≥29 on the Mood and Feelings Questionnaire (MFQ).

Figure 2 shows that at baseline, the proportion identified as having the presence of major depression (a score of ≥29 on the MFQ) was higher in the Stressbusters group than the website group. All scored >20 indicating any depressive disorder. At 4 months, the proportions were 55% (23/42) in the Stressbusters group and 68% (30/44) in the website group and at 12 months the proportions were 61% (23/38) in the Stressbusters group and 54% (20/37) in the website group.

Table 2 shows the scores for those who completed the BDI, MFQ and SCAS (both total and subscale scores) at baseline, 4 months and 12 months. Higher scores on the BDI, MFQ and SCAS represent greater levels of depressive (BDI, MFQ) or anxiety (SCAS) symptoms.

- At baseline, the mean scores attained on the MFQ was 37.5 (s.d. = 9.2) for the Stressbusters group and 35.3 (s.d. = 9.9) for the website group. In the multilevel model, mean difference in the change from baseline at 4 months was 6.3 (95% CI −1.1 to 13.7, *P* = 0.097) and at 12 months was 0.5 (95% CI −9.3 to 8.2, *P* = 0.904).
- At baseline, the mean scores attained on the BDI was 18.0 (s.d. = 6.9) for the Stressbusters group and 16.0 (s.d. = 6.6) for the website group. In the multilevel model, mean difference in the change from baseline at 4 months was 3.0 (95% CI −1.5 to 7.5, *P* = 0.192) and at 12 months was 1.5 (95% CI −3.3 to 6.3, *P* = 0.528).
- At baseline, the mean scores attained on the SCAS was 46.6 (s.d. = 15.9) for the Stressbusters group and 42.7 (s.d. = 19.0) for the website group. In the multilevel model, mean difference in the change from baseline at 4 months was 1.8 (95% CI −8.6 to 12.3, *P* = 0.728) and at 12 months −0.9 (95% CI −12.7 to 10.9, *P* = 0.8).

**Table 2:** Scores on the Mood and Feelings Questionnaire (MFQ), Beck Depression Inventory (BDI) and Spence Children's Anxiety Scale (SCAS) at baseline, 4 months and 12 months and multilevel model findings for those completing all measures

	Stressbusters						Websites						Multilevel model
	*n*	Mean	s.d.	Median	Min	Max	*n*	Mean	s.d.	Median	Min	Max	Mean difference in change from baseline (95% CI); *P*
MFQ													
Baseline	68	37.5	9.2	37.0	21.0	61.0	69	35.3	9.9	34.0	20.0	58.0	–
Month 4	42	31.5	16.4	35.5	1.0	60.0	44	35.5	15.1	39.0	3.0	61.0	6.3 (−1.1 to 13.7); 0.097
Month 12	38	31.7	19.0	32.5	0	65.0	37	29.0	16.7	33.0	4.0	53.0	0.5 (−9.3 to 8.2); 0.904
BDI													
Baseline	70	18.0	6.9	18.0	0	35.0	69	16.0	6.6	17.0	3.0	31.0	–
Month 4	42	14.4	9.3	14.5	0	34.0	44	15.4	9.2	14.5	0	34.0	3.0 (−1.5 to 7.5); 0.192
Month 12	38	12.8	9.7	11.5	0	32.0	37	12.3	8.9	12.0	0	33.0	1.5 (−3.3 to 6.3); 0.528
SCAS													
Baseline	70	46.6	15.9	45.5	10.0	82.0	69	42.7	19.0	45.0	6.0	84.0	–
Month 4	42	45.5	19.6	47.5	15.0	84.0	44	43.4	21.2	41.0	5.0	78.0	1.8 (−8.6 to 12.3); 0.728
Month 12	38	43.7	22.8	46.5	8.0	91.0	37	38.9	22.3	34.0	3.0	82.0	−0.9 (−12.7 to 10.9); 0.884

Min, minimum; Max, maximum.

### Health economic analysis results

**Fig. 3:**
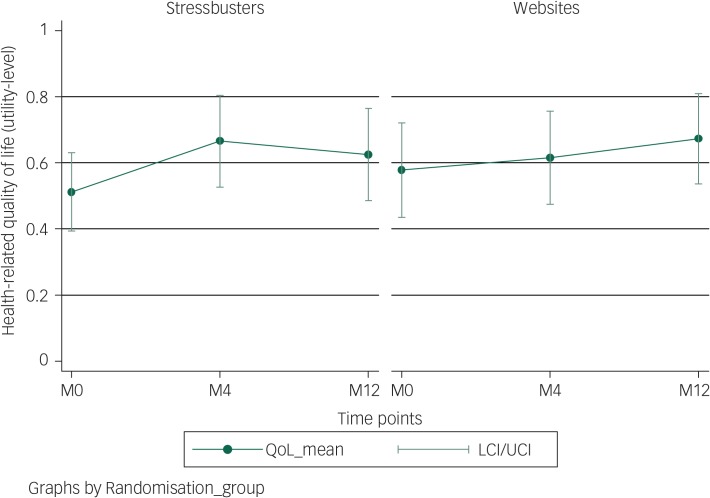
Health-related quality of life (utility QoL) levels of children in Stressbusters and websites groups during the study.

M0, baseline; M4, month 4; M12, month 12; LCI, lower confidence interval; UCI, upper confidence interval.

**Fig. 4:**
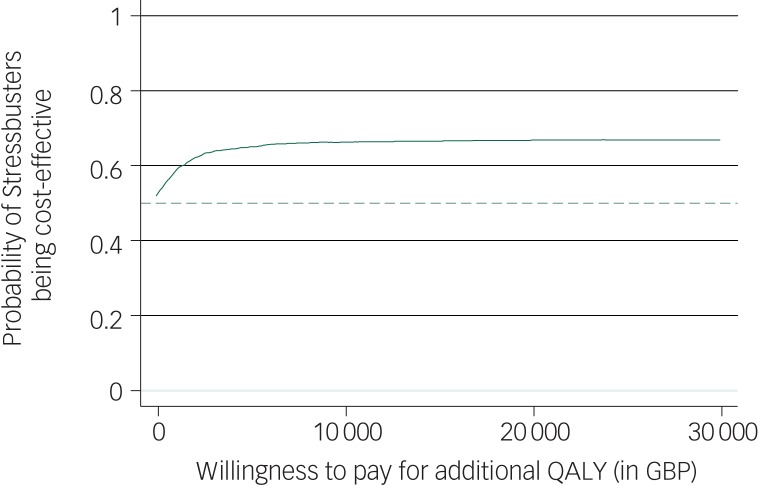
Cost-effectiveness acceptability curve for probability of Stressbusters being cost-effective at different levels of willingness to pay for an additional quality-adjusted life-year (QALY).

Complete data for all three time points was available for 51 children (Stressbusters group  27; websites group 24). Figure 3 shows that both groups showed improvement in QoL (utility) over time with the Stressbusters group showing early gain compared with the website group; however, the CIs overlapped. QALYs over the follow-up period was 0.622 (95% CI  0.514–0.729) for Stressbusters and 0.623 (95% CI 0.521–0.725) for the websites group. After adjusting for difference in baseline utility, the difference in utility (Stressbusters minus websites) was 0.029 (95% CI  −0.09 to 0.14) and was not statistically significant (Table 3). Costs, including the intervention cost, were lower in the Stressbusters group compared with the website group but the difference was not statistically significant (−£14.9, 95% CI −£246.0 to £216.2) (Table 3). The ICER was negative (and therefore not reported) because of lower costs and higher QALYs in the Stressbusters group. The CEAC showed that there was just over 65% probability that Stressbusters is cost-effective if the WTP is between £20 000 and £30 000 per QALY (Fig. 4).

GBP, Great British pounds.

**Table 3:** Regression result for difference in costs and quality-adjusted life-years (QALYs) after controlling for baseline utility

Variables	Coefficient	Standard error (bootstrap)	*z*	*P*>*z*	95% CI
Difference in cost (Stressbusters minus website group), £	−14.90	117.91	−0.13	0.90	−245.99	216.19
Difference in QALYs (Stressbusters minus website group)	0.03	0.06	0.49	0.63	−0.09	0.14

## Discussion

### Main findings

Overall scores on outcome measures were not significantly different between the intervention and attention control groups at 12 months, however, both showed improvements on all measures. This may be because many young people improve as time progresses^27^ or because the website intervention yielded benefits to the young people that were just as helpful as CCBT.

### Implications

This finding was the same for QoL measures, both groups showed improvement in QoL (utility) over time. Interestingly, although the Stressbusters group showed earlier improvement compared with the website group^11^ the difference reduced over time. This could suggest that early gains from CCBT are then subsequently ‘caught up’ by appropriate depression self-help accessibility or that the natural course of depression in teenagers is variable but remitting in many young people. The other published CCBT Stressbusters study in adolescent depression^9^ suggests that CCBT is more efficacious compared with waiting list control over 6 months (they did not follow-up for 1 year). These trials together present the interesting suggestion that as websites are free to use and that the outcomes after a year are promising a stepped-care approach may be helpful involving options on the waiting list such as self-help website access followed by CCBT that then leads on to face-to-face therapy such as CBT or interpersonal therapy. A larger RCT would need to be planned to examine the clinical and cost-effectiveness of this promising idea. Any such trial would need to carefully examine researcher or other effects and mechanisms more closely.

Costs, including the intervention cost, were lower in the Stressbusters group compared with website group but again the difference was not statistically significant. Taking costs and outcomes together, there is just over 65% chance of Stressbusters being a cost-effective intervention compared with well-chosen self-help websites. This reflects significant uncertainty in both costs and outcomes data, and suggests that, although there is some indication supporting the economic value of stressbusters, further research is required to resolve uncertainty in evidence before services should consider adapting this technology in routine practice.

This suggests that both CCBT and well-chosen self-help websites provide benefit for young people and given no serious adverse events, that these interventions may have a place in the care pathway. The current National Institute for Health and Care Excellence guidelines in the UK for treating adolescent depression suggest that those with moderate-to-severe depression should be offered a talking therapy that could be individual CBT, interpersonal therapy, family therapy or psychodynamic psychotherapy.^28^ Many young people are currently having to wait long periods of time for this.^29^ A London School of Economics report cited various research estimating that only 25% of young people with mental health conditions access treatment and in a survey of 590 general practitioners only 6% could access treatment within 2 months when they had a child they felt needed specialist psychological therapy beyond counselling.^30^ A total of 78% reported being rarely able to access this support. Reduced access to treatment could be related to limited recognition, reluctance to come forward, stigma and limited service availability. This suggests that adding more options to the care pathway may be helpful in increasing access to early treatment, particularly earlier on in the care pathways and in accessible places such as schools. Given promising findings for both CCBT and self-help websites, further research on stepped-care models and treatments in easy to access places, that are alternatives to face-to-face treatment and medication, should be undertaken.

### Strengths and limitations of this study

The study uses a range of mental health and health economics measures in a population of adolescents with low mood. Rich information regarding the application of CCBT as a treatment for this group in the community (notably in schools) has been yielded through this study. Those involved in the trial including adolescents, their parents and CAMHS clinicians were largely supportive of CCBT as a treatment for adolescent depression with steady recruitment throughout the trial period. In addition, as part of the trial we developed a strong infrastructure comprising 10 schools, 2 clinics, 1 general practice and 1 community centre in which we delivered the trial.

Despite the strengths of the study, numerous challenges were also faced and present throughout all stages of the trial including its setup, recruitment period and delivery.^31^ As the trial was only being delivered within one NHS trust certain minorities were underrepresented and there was a lack of varied geographical localities and demographic characteristics. As discussed in the 4-month outcome paper,^11^ a larger RCT would need to be extended geographically to include a wider ethnic and sociocultural diversity.

We set out to measure time spent on CCBT and websites using a commercial software package. However, this data is not reported as it was very unreliable because of a software malfunction. Follow-up numbers were lower than hoped for at the 12-month follow-up point but in line with other research including young people with depression.^32^ This means that results should be treated with caution as some post-treatment results (and possible group differences) are not known.

The cost of the Stressbusters program (£101.20 per participant) did not include costs for any upgrades required. This would need to be considered in any future trial. We have summarised and published the lessons we learned from planning and conducting this RCT in a separate paper.^31^ That paper provides further details about how the issues we faced were resolved by the research team using a variety of techniques.
